# Medication Management of Patients With Cancer Undergoing Surgery From Preadmission to Discharge: A Mixed‐Methods Systematic Review

**DOI:** 10.1111/jan.16759

**Published:** 2025-01-21

**Authors:** Mehrabifar Atefeh, Manias Elizabeth, Nicholson Patricia

**Affiliations:** ^1^ School of Nursing and Midwifery, Centre for Quality and Patient Safety Research Institute for Health Transformation, Deakin University Geelong Victoria Australia; ^2^ School of Nursing and Midwifery, Faculty of Medicine, Nursing and Health Sciences Monash University Melbourne Victoria Australia

**Keywords:** cancer, collaboration, communication, discharge, medication errors, medication therapy management, perioperative, polypharmacy, preadmission, surgery

## Abstract

**Aim(s):**

To identify and synthesise available evidence about regular medication management processes, from preadmission to discharge from hospital, in patients with cancer undergoing surgery.

**Design:**

Mixed‐methods systematic review.

**Methods:**

Studies published from inception of each database until February 2023 were screened, utilising four main search concepts. The JBI methodology for mixed‐methods systematic review was followed in this review.

**Data Sources:**

MEDLINE, CINAHL, EMBASE, APA PsycINFO, Scopus and Web of Science.

**Results:**

Eight out of 717 screened studies were included. Two themes related to patients' medication management were identified. Preoperative factors such as polypharmacy, potentially inappropriate medications, delirium‐inducing medications and preoperative discontinuation‐requiring medications were associated with several postoperative complications in patients with cancer. Additionally, pharmacist‐led interventions and collaborative efforts between nurses and patients were shown to improve the medication management process across the perioperative pathway.

**Conclusion:**

This systematic review emphasises the necessity of effectively managing regular medication, especially before surgery, to mitigate postoperative complications in patients with cancer. It offers critical insights into how involving pharmacists and nurses enhances medication management outcomes, benefiting health care professionals and institutions aiming to optimise perioperative medication therapy.

**Implications for the Profession and/or Patient Care:**

Enhancing patients' regular medication management through comprehensive reviews before surgery, and improving collaborative practices among pharmacists, nurses and patients via targeted interventions introduced by health care organisations, ensure safe medication use throughout the perioperative pathway.

**Impact:**

Improving regular medication management process can reduce risk of medication errors and adverse drug events and enhance postoperative outcomes.

**Reporting Method:**

SWiM reporting guidelines.

**Patient or Public Contribution:**

No patient or public contribution.

**Summary**

- What does this paper contribute to the wider global clinical community?
  ○ Optimising the process of patients' preoperative medication use can mitigate the risk of postoperative complications, thus reducing significant health care costs.
  ○ According to the definition of medication management, multidisciplinary collaboration—including collaborative efforts and communication between patients and nurses, as well as pharmacist‐led interventions—is a crucial aspect that merits more attention, particularly for patients with cancer when undergoing surgery.

## 1 Introduction

Cancer remains one of the most significant health challenges worldwide, accounting for nearly one in six deaths (Debela et al. 2021). Surgical interventions continue to be a cornerstone in the treatment and management of most solid organ cancers, playing a crucial role in both curing cancer by removing localised tumours and in controlling the disease by reducing tumour burden (Tohme, Simmons, and Tsung 2017). Despite advances in surgical oncology, managing the concurrent health conditions and pharmacological needs of cancer patients remains a critical aspect of care, particularly in the perioperative setting.

Evidence shows about 40% of patients with cancer have at least one comorbidity and are prescribed regular medications to manage their overall health (Sarfati, Koczwara, and Jackson 2016). Notably, the presence of comorbidities has been shown to significantly impact cancer patients' clinical outcomes, often complicating treatment plans and necessitating tailored approaches to regular medication management (Sarfati, Koczwara, and Jackson 2016). Among the complexities of cancer care, effective management of patients' regular medications throughout the perioperative period is paramount for ensuring optimal patient outcomes (Jasper, Dhesi, and Partridge 2023). However, this task is not without its challenges.

Multidisciplinary experts' involvement, the necessity of fasting before and after surgery and the absence of clear guidelines represent critical hurdles in managing regular medications across the perioperative pathway (Russell et al. 2022). The intricate interplay between cancer, surgical procedures and pharmacotherapy used to treat comorbidities necessitates a comprehensive understanding of the challenges and opportunities inherent in medication management process across the perioperative continuum (Flynn et al. 2020).

Surgical patients experience alterations in regular medication regimens because of the surgical intervention, the presence of comorbidities and multiple in‐hospital transfers, which can significantly impact their postoperative outcomes. Poor medication management can lead to postoperative complications, prolonged hospital stays and increased risk of readmission (de Boer et al. 2013; He et al. 2015), especially among patients with cancer for whom these risks are exacerbated by the narrow therapeutic margins of antineoplastic agents and the concurrent use of multiple regular medications (Kefale et al. 2022).

Current studies have highlighted the importance of medication review and multidisciplinary collaboration to improve perioperative outcomes. Latimer et al. (2023) and Nilsson, Gruen, and Myles (2020) underscored the necessity of improved communication and collaborative efforts among health care professionals in the decision‐making process, while Guisado‐Gil et al. (2021) further demonstrated that implementing a preoperative medication review programme can significantly reduce hospital stays after surgery. Similarly, Falconer et al. (2022) reported that postoperative pharmacist reconciliation services led to a significantly lower readmission rate in the postintervention group compared to the preintervention group. Nevertheless, these efforts remain fragmented, with substantial variability in practices across health care systems. Furthermore, while the impact of medication management on general surgical populations has been explored, there is limited evidence specific to cancer patients, who often present with more complex clinical profiles. This systematic review aims to bridge these gaps by critically evaluating the current literature on perioperative medication management for cancer patients. By systematically analysing available evidence, we seek to elucidate current practices, identify gaps in knowledge and propose recommendations for optimising medication management strategies in this complex patient population. Such insights have the potential to inform clinical guidelines, enhance multidisciplinary collaboration and ultimately improve outcomes for cancer patients undergoing surgery.

## 2 The Review

While several systematic reviews have addressed perioperative medication management, including pain‐ and infection‐related medication management (An et al. 2022; Flynn et al. 2020; Iocca et al. 2022), a comprehensive review specifically focused on the challenges of regular medication management in surgical patients with cancer has not been conducted. To address this, a systematic review protocol was developed and submitted to the PROSPERO database (Centre for Review and Dissemination) on 1st of December 2022. In this systematic review, patients' medications related to their comorbidities are referred to as regular medications.

## 3 Aim

This systematic review aimed to comprehensively synthesise the existing literature pertaining to the management of regular medications in patients with cancer undergoing surgery from preadmission to discharge.

## 4 Methods/Methodology

### 4.1 Design

A mixed‐methods approach was employed, following the JBI methodology (Lizarondo et al. 2019), integrating findings from studies that utilised either or both qualitative and quantitative data.

### 4.2 Search Methods

A comprehensive search strategy was developed using PRISMA guidelines (Page et al. 2021). During the first stage, a preliminary search was conducted in Google Scholar to identify keywords used in the title and abstract of the articles. A systematic and comprehensive search was then conducted of different databases, including MEDLINE Complete via Ebsco, CINAHL Complete via Ebsco, EMBASE via EMBASE and APA PsycINFO via Ebsco. The detailed search strategy of the included databases can be found in the Appendices [Link], [Link], [Link], [Link] online (URL link will be provided prior to publication). The next step involved conducting a snowball search of key articles, where references and citations in Scopus and Web of Science were cross‐checked by one reviewer to retrieve relevant articles. Furthermore, a manual review of the reference lists of all included studies was conducted by the reviewer to identify any additional relevant studies. If any reviews were identified, the included studies were manually screened to assess their eligibility based on the criteria. The searches covered four main concepts, including ‘medication management’, ‘surgery’, ‘cancer patients’ and ‘preadmission to discharge’.

### 4.3 Inclusion and/or Exclusion Criteria

All study types, including prospective, longitudinal, retrospective, cross‐sectional, case–control, cohort, qualitative and mixed‐methods studies were eligible for inclusion. No date restrictions were applied. Only English articles were included in this systematic review. Studies conducted on adult patients aged 18 years and over, diagnosed with cancer and undergoing surgery, were included. Reviews, opinion papers, conference abstracts and grey literature were excluded. Studies were excluded if they focused on paediatric and/or adolescent populations. Studies focused on day surgery were excluded. A number of studies were conducted in both surgical and medical settings. These studies were excluded from the review when findings specific to the surgical settings were not reported. Studies that involved conduct of comprehensive assessments of cancer patients prior to surgery but did not primarily address medication management were excluded from this review. Therefore, all articles published from the inception of each database to November 2022 were initially screened, with updates made in February 2023.

### 4.4 Search Outcome

**FIGURE 1:**
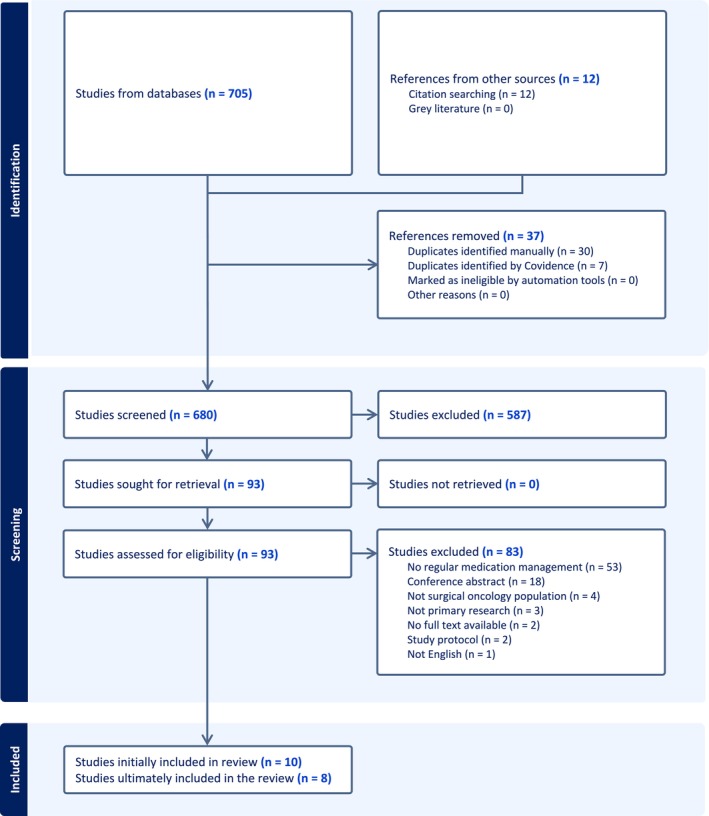
PRISMA 2020 flow diagram for the selection of studies (Page et al. 2021).

The bibliographic software, EndNote version 20, was used to record and manage data, where duplicates were removed. Subsequently, Covidence software was employed to further eliminate duplicates and to screen articles in abstracts, title and full‐text levels by two independent reviewers. Any disagreement in inclusion or exclusion of a study was resolved through negotiation of two reviewers, and if necessary, the third independent reviewer checked the issue and resolved it through consensus. A total of 717 articles were retrieved, including 12 articles found in snowball search. After removing 37 duplicates, 680 articles were screened at abstract and title level. Ninety‐three articles were reviewed for eligibility at full‐text level, with 10 studies initially included. Subsequently, two studies were excluded due to the primary aim of the study not focused on regular medication management. Ultimately, eight studies (*n* = 7 quantitative; *n* = 1 qualitative) were included in this systematic review as shown in Figure 1, the PRISMA 2020 flow diagram (Page et al. 2021).

### 4.5 Quality Appraisal

For quantitative studies, methods of randomisation, data collection process and results were appraised while the methodology and research findings of the qualitative studies were evaluated using a quality assessment tool. Two reviewers evaluated the quality of the studies using the Mixed Methods Appraisal Tool (MMAT), Version 2018, which is designed to assess both quantitative and qualitative studies (Hong et al. 2018). A third reviewer checked the two reviewers' decisions. Overall, the studies met the quality criteria, with few areas of concern which is shown in Table 1, Quality Appraisal (Hong et al. 2018). The research questions in the studies were well defined and clear, and the collected data addressed the research questions. As for the quantitative studies, participants were representative of the target population. Confounders were reasonably taken into consideration in the design and analysis of the studies. Regarding the survey conducted by Triscari, Teoh, and Femia (2022), the inclusion and exclusion criteria for patients and health care professionals were not explained in the article. Additionally, the authors did not mention the method of recruitment, piloting the questionnaire, response rate and the characteristics of the respondents and nonrespondents.

**TABLE 1:** Quality appraisal.

	Quantitative, nonrandomised						
	Screening questions		Quality assessment				
Author, Publication date	Are there clear research questions?	Do the collected data allow to address the research questions?	Are the participants representative of the target population?	Are measurements appropriate regarding both the outcome and intervention (or exposure)?	Are there complete outcome data?	Are the confounders accounted for in the design and analysis?	During the study period, is the intervention administered (or exposure occurred) as intended?
Chen et al. (2021)	Yes	Yes	Yes	Yes	Yes	Yes	Yes
Choi et al. (2018)	Yes	Yes	Yes	Yes	Yes The section concerning the three patients who died within 30 days after surgery would benefit from additional elaboration, particularly with regard to key factors such as polypharmacy. Nevertheless, it is important to note that the researchers acknowledged the limitation of a low number of deaths and insufficient data for conducting a meaningful analysis.	Yes	Yes
Jeong et al. (2018)	Yes	Yes	Yes	Yes	Yes	Yes	Yes
Jeon et al. (2020)	Yes	Yes, to some extent. While the collected data partially enable addressing the research question, the retrospective nature of the study imposes limitations. It hinders the ability to definitively pinpoint the root cause of readmissions. Consequently, the researchers lacked confidence in determining whether readmissions were drug‐related or not, thereby impeding the assessment of preventability. It is important to note that the authors acknowledge this study's limitation.	Yes	Yes	Yes	Yes	Yes
Jeong et al. (2016)	Yes	Yes	Yes	Yes	Yes	Yes	Yes
Samuelsson et al. (2016)	Yes	Yes	Yes	Yes	Yes	Yes	Yes

	Survey						
	Screening questions		Quality assessment				
Author, Publication date	Are there clear research questions?	Do the collected data allow to address the research questions?	Is the sampling strategy relevant to address the research question?	Is the sample representative of the target population?	Are the measurements appropriate?	Is the risk of nonresponse bias low?	Is the statistical analysis appropriate to answer the research question?
Triscari, Teoh, and Femia (2022)	Yes	Yes	No	No (Number of staff working in PAC not reported)—only six patient surveys were conducted (178 were seen by pharmacist)	No (no details about piloting the survey provided)	No (low response rate from patients)	Can't tell

	Observation and interview						
	Screening questions		Quality assessment				
Author, Publication date	Are there clear research questions?	Do the collected data allow to address the research questions?	Is the qualitative approach appropriate to answer the research question?	Are the qualitative data collection methods adequate to address the research question?	Are the findings adequately derived from the data?	Is the interpretation of results sufficiently substantiated by data?	Is there coherence between qualitative data sources, collection, analysis and interpretation?
Uhrenfeldt and Høybye (2014)	Yes	Yes	Yes	Yes	Yes	Yes	Yes

### 4.6 Data Extraction

Two reviewers independently extracted study details using a modified Cochrane data collection form template tailored to specific data requirements of the review (Glenton et al. 2022). The third reviewer checked the extracted data and investigated and resolved any conflicts between the two reviewers through consensus. The data extracted are presented in Table 2, Data extraction (Glenton et al. 2022).

**TABLE 2:** Data extraction.

Author, publication date, country	Study design	Aim	Sample, setting	Outcome measures	Findings
Chen et al. (2021), Germany	Retrospective cohort study (Quantitative nonrandomised)	To evaluate the association between polypharmacy (use of five or more medications) and 5‐year cancer survival	Patients with colorectal cancer aged 65 years and older from 22 hospitals in Southwestern Germany. Mean age = 75.0 years (SD = 6.5 years) Female = 41%	Overall survival up to 5 years = duration from admission to hospital until death Cancer‐specific survival = duration from admission to hospital until death due to cancer Non‐cancer‐specific survival = duration from admission to hospital until death resulting from causes other than cancer	Increasing number of medications with poorer overall survival (HR = 1.23; 95% CI 1.02–1.47; *p*‐value < 0.05). Using more than eight medications with colorectal cancer‐specific survival (HR = 1.31; 95% CI 1.03–1.68), and non‐cancer‐specific survival (HR = 2.15; 95% CI 1.62–2.87).
Choi et al. (2018), South Korea	Retrospective analysis of prospectively collected data (quantitative nonrandomised)	To evaluate the effect of preoperative medications on postoperative morbidity and mortality	Patients with cancer, aged 65 years or older who underwent surgery and a comprehensive geriatric assessment in the Geriatric Centre of Seoul National University Bundang Hospital. Median age = 76 years (range 65–96) Females = 55% Patients mostly had gastrointestinal cancers (72%) and breast cancer (20%)	Postdischarge institutionalisation and death within 30 days after surgery	Three patients using polypharmacy died within 30 days postoperatively. Increasing polypharmacy with higher probability of postdischarge institutionalisation (AOR = 3.96; 95% CI 1.05–14.86; *p*‐value < 0.05) Using potentially inappropriate medication with higher risk of mortality (*p*‐value = 0.12) Using potentially inappropriate medication with higher risk of postdischarge institutionalisation (OR = 0.76; 95% CI 0.21–2.78; *p*‐value = 0.65)
Jeong et al. (2018), South Korea	Retrospective cohort study (quantitative nonrandomised)	To explore the association between preoperative medication use and postoperative length of hospital stay (LOS)	Patients aged 65 years and older who underwent elective cancer surgery and preoperative comprehensive geriatric assessment in the Geriatric Centre of Seoul National University Bundang Hospital. Median age = 76 years (range 65–96) Patients aged 75 years and older = 59.2% Female = 55% Patients mostly had gastrointestinal cancers (72%) and breast cancer (20%)	Assessing the length of hospital stay after cancer surgery	Cancer type (parameter estimate = −4.43; *R* ^2^ value = 0.09; *p*‐value < 0.001), increasing number of medications (parameter estimate = 0.20; *R* ^2^ value = 0.01; *p*‐value = 0.02), and using potentially inappropriate medications (parameter estimate = 1.60; *R* ^2^ value = 0.02; *p*‐value = 0.03) with increased length of stay. Use of potentially inappropriate medications associated with a hospital stay 1.6 times longer in multiple linear regression analysis after adjustment for potential confounding factors. Using preoperative discontinuation requiring medication with increased length of hospital stay (*p*‐value = 0.65).
Jeon et al. (2020), South Korea	Retrospective analysis of prospectively collected data (quantitative nonrandomised)	To evaluate the association of preoperative medication use with hospital readmission within 30‐day postoperatively	Patients aged 65 years or older who underwent elective cancer surgery and a comprehensive geriatric assessment in the Geriatric Centre of Seoul National University Bundang Hospital. Median age = 76 years (range 65–96). Patients aged 75 years or older = 59.2% Female = 55% Patients mostly had gastrointestinal cancers (72%) and breast cancer (20%)	Unplanned readmission within 30 days postoperatively.	Preoperative discontinuation‐requiring medication use (medications that should be stopped prior to surgery due to surgical risks) with increasing risk of readmission (AOR = 2.18; 95% CI 1.01–4.70; *p*‐value < 0.05). Taking herbal extracts with increasing risk of unplanned 30‐day readmission (OR = 2.65; 95% CI 1.03–6.79; *p*‐value = 0.05).
Samuelsson et al. (2016), Sweden	Retrospective analysis of prospectively recorded data (quantitative nonrandomised)	To explore the association of the continuation of potentially inappropriate medication (PIM) with the length of hospital stay and postoperative mortality	Individuals aged 75 years or older who underwent colorectal cancer surgery in Sweden from 2007 to 2010. Mean age in PIM‐exposed group = 81.8 Mean age in nonexposed group = 81.2 Females in PIM‐exposed group = 61% Females in nonexposed group = 50%	The length of stay in hospital, death within 30 days following surgery	Extended length of stay with using potential inappropriate medication (OR = 1.14; 95% CI 1.00–1.29; *p*‐value = 0.05) Patients of the potentially inappropriate medication group were more likely to being discharged to other institutions (20.2% vs. 14.5%, *p*‐value < 0.001). Using potentially inappropriate medications with higher mortality rate (OR = 1.43; 95% CI 1.11–1.85; *p*‐value = 0.01).
Jeong et al. (2016), South Korea	Retrospective cohort study (quantitative nonrandomised)	To examine the association between preoperative medication use and postoperative delirium	Patients aged 65 years and older who underwent elective cancer surgery and preoperative comprehensive geriatric assessment in the Geriatric Centre of Seoul National University Bundang Hospital. Median age = 76 years (range 65–96) Patients aged 75 years and older = 59.2% Females = 55% Patients mostly had gastrointestinal cancers (72%) and breast cancer (20%)	Postoperative delirium	An additional year of age associated with approximately 1.1‐fold increase in the risk of postoperative delirium. Delirium‐inducing medication use with increasing risk of postoperative delirium (AOR = 12.78; 95% CI 2.83–57.74; *p*‐value < 0.001) Potentially inappropriate medication use with increasing risk of postoperative delirium (AOR = 5.53; 95% CI 2.03–15.05; *p‐*value < 0.001) There was no association between preoperative discontinuation‐requiring medications and postoperative delirium (Unadjusted OR = 1.03; 95% CI 0.41–2.60; *p‐*value = 1.00).
Triscari, Teoh, and Femia (2022), Australia	Prospectively recorded data (quantitative survey)	To assess the pharmacists' services provided for health care professionals and patients at the preadmission clinic	Patients attending the preadmission clinic at the maternity and gynaecological hospital in Western Australia were invited to complete a survey. Additionally, an online survey was shared with health care professionals working in the preadmission clinic. Response rate: anaesthetists (*n* = 8) and nurses or midwives (*n* = 7).	Satisfaction and added value provided by the PAC pharmacists from both the multidisciplinary team and patients' viewpoints. Performance metrics recorded by the PAC pharmacists. * A scale ranging from 1 to 5 was utilised, with 1 representing the least agreeable and 5 indicating the most agreeable.	Staff members rated pharmacists helped to reduce medication errors on admission: 27% agreed, 60% strongly agreed and 13% selected neutral option. In relation to the statement ‘pharmacists contribute to acquiring a more precise medication history’, 73% strongly agreed, and 27% expressed agreement. Patient understanding of pharmacist role at PAC (increase from 4 to 4.67 out of 5), medication comprehension (increase from 4.17 to 4.50 out of 5), confidence in medication changes (4.83 out of 5) and overall satisfaction (4.83 out of 5) improved postinterviews. Medications recommenced after withholding, those necessitating alterations to therapy before a procedure, and medications prompting patient queries identified as the most important medications subject to clinical interventions.
Uhrenfeldt and Høybye (2014), Denmark	Ethnographic study (observation and interview)	To investigate the challenges encountered by older surgical patients during a short hospital stay	Patients aged 74 years and older, diagnosed with colon cancer, who underwent surgery and encountered challenges, such as oral health and polypharmacy during their admission at a Danish teaching hospital. *N* = 9 (aged between 74 and 84 years) Males = 4 Females = 5	—	Older individuals who were observed with trembling hands or impaired vision may struggle to retrieve tablets from a cup, leading to potential medication loss and uncertainty in administration process. Individuals with impaired hearing, despite using a hearing aid, encountered challenges assisted with medication consumption. Dry mouth, known as xerostomia, primarily occurs as a result of systemic medications, posing a heightened risk for older individuals who frequently take more medication.

* Indicates the scale used for evaluation, with 1 representing the least agreeable and 5 representing the most agreeable.

### 4.7 Study Risk of Bias Assessment

To minimise potential bias, several strategies were employed. Initially, the search strategy was developed in consultation with a specialist health librarian. Subsequently, two reviewers independently conducted screening, with conflicts resolved by a third reviewer. Quality appraisal and data extraction were performed independently by the two reviewers, and overseen by the third reviewer. Collective decision‐making was integral throughout, with regular meetings to review progress and determine next steps.

### 4.8 Synthesis

Due to the varied nature of the study designs, objectives and outcomes, a synthesis without meta‐analysis (SWiM) approach (Campbell et al. 2020) was used to summarise and present a comprehensive analysis of quantitative findings. The quantitative studies were grouped into themes based on the exposure variables and outcome measured. A synthesis without meta‐analysis was developed by assessing the consistency of findings and the relevance of measured variables to the review question. Certainty of the results was considered through careful comparison of participant characteristics, incidence of outcome occurrences, and evaluation of potential confounding factors across the included studies. For the qualitative study included in the systematic review, a narrative synthesis was conducted to identify key themes and insights related to the research question, utilising the framework approach outlined by Braun and Clarke (2022). The study was read line by line to identify and code the findings relevant to the review question. Following, the qualitative insights were used to contextualise and explain the quantitative results where possible, providing a deeper understanding of the mechanisms underlying the observed outcomes.

## 5 Results/Findings

### 5.1 Characteristics of Included Studies

Of the eight studies included in the review, seven utilised a quantitative design and one used a qualitative design. Four studies were conducted in South Korea, and one each in Germany, Sweden, Australia and Denmark. Four studies conducted in South Korea utilised the baseline information from the same participants through secondary analysis to investigate various outcomes (Choi et al. 2018; Jeon et al. 2020; Jeong et al. 2016, 2018). The number of participants taking part in each study ranged from 9 to 7279 in included studies. Six studies employed a retrospective analysis of a prospectively collected data design (Chen et al. 2021; Choi et al. 2018; Jeon et al. 2020; Jeong et al. 2016, 2018; Samuelsson et al. 2016), one utilised a quantitative survey (Triscari, Teoh, and Femia 2022) and the remaining qualitative study employed observational methods combined with interviews (Uhrenfeldt and Høybye 2014). Seven studies focused on geriatric populations aged 65 years and older.

### 5.2 Review Findings

The included studies were synthesised based on two primary themes: (1) Medication use and postoperative complications, and (2) Evaluation of interventions within the medication management process. The findings reveal the multifaceted nature of medication management in cancer patients undergoing surgery, highlighting both the challenges and opportunities for improvement. The first theme explores how factors such as polypharmacy and specific medication categories impact postoperative outcomes, while the second theme focuses on the effectiveness of various interventions, such as pharmacist‐led initiatives, in mitigating medication‐related risks. Together, these themes underscore the critical role of structured and collaborative medication management practices in enhancing surgical outcomes for cancer patients. By addressing both the risks associated with medication use and the benefits of targeted interventions, the findings offer valuable insights to inform clinical practice and improve surgical outcomes for cancer patients.

#### 5.2.1 Medication Use and Postoperative Complications

It is evident that preoperative medication management was associated with postoperative complications, including postdischarge institutionalisation, prolonged hospital length of stay, postoperative delirium, readmissions and mortality within 30 days of surgery in cancer patients (Chen et al. 2021; Choi et al. 2018; Jeon et al. 2020; Jeong et al. 2016, 2018; Samuelsson et al. 2016). To analyse the findings from the studies included in this systematic review, two subthemes are elucidated.

##### 5.2.1.1 Polypharmacy and Its Implications

When defining polypharmacy, five studies clearly specified the types of medications included in the counting process, with all of them considering the use of five or more medications as polypharmacy (Chen et al. 2021; Choi et al. 2018; Jeon et al. 2020; Jeong et al. 2016, 2018). Excessive polypharmacy was defined as the concurrent use of eight or more medications by Chen et al. (2021), while the other four studies defined it as using 10 or more medications (Choi et al. 2018; Jeon et al. 2020; Jeong et al. 2016, 2018). Four studies used the same criteria for counting patients' regular medications, while one study used a modified set of criteria. Supplements, herbal remedies and topical medications were excluded in Chan et al.'s study, whereas the other four studies included all these types of medications, except for topical medications. Additionally, Chen et al. (2021) specifically excluded three common categories of postsurgery medications when counting the total number of patients' medications reported in the discharge letter: antithrombotic agents, nonsteroidal anti‐inflammatory drugs and peptic ulcer disease treatments. These medications were excluded because they were intended for short‐term use following surgery and not for treating comorbidities.

Overall, the prevalence of polypharmacy in the studies ranged from 51% to 55%. This high percentage is likely related to the older participants (aged ≥ 65 years), who were more prone to comorbidities and the need for multiple regular medications. Additionally, four out of five studies, focusing on polypharmacy, included the same cohort of patients (Choi et al. 2018; Jeon et al. 2020; Jeong et al. 2016, 2018), with conflicting results regarding the impact of polypharmacy on postoperative complications reported. In a retrospective cohort study of 475 cancer patients, polypharmacy was significantly associated with postdischarge institutionalisation, defined as postsurgery transfer to other facilities (AOR = 3.96; 95% CI 1.05–14.86; *p*‐value < 0.05). The wider confidence interval observed in the association between polypharmacy and postdischarge institutionalisation may be attributed to the limited number of outcome events (14 events of postdischarge institutionalisation among 475 patient). Additionally, there were insufficient data to establish an association with postoperative mortality as only three deaths were reported (Choi et al. 2018). Another study involving the same participants showed a marginally significant association between excessive polypharmacy and prolonged hospital stay (parameter estimate = 0.20; *R* ^2^ value = 0.01; *p*‐value = 0.02) (Jeong et al. 2018). Likewise, excessive polypharmacy showed a significant association with poorer cancer survival in patients with colorectal cancer aged 65 years and older (HR = 1.23; 95% CI 1.02–1.47; *p*‐value < 0.05) (Chen et al. 2021). In two retrospective cohort studies involving the same participants, polypharmacy was examined for its association with postoperative delirium (OR = 2.19; 95% CI 0.82–5.85; *p*‐value = 0.16) (Jeong et al. 2016), and unplanned 30‐day readmission (*p*‐value = 0.66) (Jeon et al. 2020), with neither study reporting statistical significance.

##### 5.2.1.2 Regular Medication Categories

Several studies focused on different categories of patients' regular medications and postoperative complications (Choi et al. 2018; Jeon et al. 2020; Jeong et al. 2016, 2018; Samuelsson et al. 2016). Most studies found that potentially inappropriate medications had a significant association with postoperative complications. Potentially inappropriate medications are those where the risk of adverse events surpasses the clinical benefits, especially when there are safer alternatives (Tamura et al. 2012). Two retrospective cohort studies conducted in 2016 and 2018 reported significant association of potentially inappropriate medication use with prolonged hospital stay ([parameter estimate = 1.60; *R* ^2^ value = 0.02; *p*‐value = 0.03 in Jeong et al.'s (2018) study] and [OR = 1.14; 95% CI 1.00–1.29; *p*‐value = 0.05 in Samuelsson et al.'s 2016 study]). Samuelsson et al. (2016) concluded that using potentially inappropriate medication significantly increased postoperative mortality risk (OR = 1.43; 95% CI 1.11–1.85; *p*‐value = 0.01). However, Choi et al. (2018) reported that using potentially inappropriate medication was not associated with postoperative mortality (*p‐*value = 0.12) and postdischarge institutionalisation (OR = 0.76; 95% CI 0.21–2.78; *p‐*value = 0.65). Moreover, Jeong et al. (2016) reported a significant association between potentially inappropriate medication use and postoperative delirium (AOR = 5.53; 95% CI 2.03–15.05; *p‐*value < 0.001).

There have been conflicting results about the association of preoperative discontinuation‐requiring medications with postoperative complications. Preoperative discontinuation‐requiring medications are medications that need to be stopped before surgery because of potential surgical risks, such as antithrombotic agents and herbal medications (Jeong et al. 2016). In three studies involving the same cohort of patients, researchers showed that preoperative discontinuation‐requiring medications had no significant association with postoperative mortality (*p*‐value = 0.40), postdischarge institutionalisation (unadjusted OR = 0.99; 95% CI 0.34–2.90; *p‐*value = 0.99) (Choi et al. 2018), prolonged hospital stay (*p‐*value = 0.65) (Jeong et al. 2018) and postoperative delirium (unadjusted OR = 1.03; 95% CI 0.41–2.60; *p‐*value = 1.00) (Jeong et al. 2016). Conversely, Jeon et al. (2020) demonstrated that preoperative discontinuation‐requiring medications, including herbal extracts, had a statistically significant association with unplanned readmissions (AOR = 2.18; 95% CI 1.01–4.70; *p‐*value < 0.05). Among the four studies examining the association between delirium‐inducing medications and postoperative complications (Choi et al. 2018; Jeon et al. 2020; Jeong et al. 2016, 2018), only one study (Jeong et al. 2016) found a significant link specifically between these medications and postoperative delirium (AOR = 12.78; 95% CI 2.83–57.74; *p‐*value < 0.001).

#### 5.2.2 Evaluation of Interventions Within the Medication Management Process

Triscari, Teoh, and Femia (2022) evaluated preadmission clinic pharmacist services by conducting a feedback survey among patients undergoing surgery and staff working in the preadmission clinic of a gynaecological oncology centre. The majority of the staff expressed strong agreement regarding the effectiveness of pharmacist interventions in mitigating medication errors (87%), obtaining an accurate medication history (73%) and resolving questions about patient medications (87%). Nurses stated that pharmacists' presence during preadmission visit enhanced their knowledge and understanding of medication. Additionally, patients reported an improvement in their understanding of medications and felt more confident about medication changes following their preadmission clinic visit. However, the low response rate suggests that the findings may not fully represent the perspectives of patients and nurses in the preadmission clinic. The pharmacist's intervention primarily targeted medications that needed to be discontinued or adjusted before the procedure, with approximately 67% of the high‐risk patients being seen at the preadmission clinic (Triscari, Teoh, and Femia 2022). Likewise, a qualitative study focused on the challenges when older patients were hospitalised for cancer surgery, including medication consumption challenges (Uhrenfeldt and Høybye 2014). Considering the physical limitations associated with older patients, especially cancer patients with specific needs, collaboration between patients and nurses was highly beneficial in optimising patients' medication consumption. For instance, the researchers observed that older patients with trembling hands and impaired vision struggled to handle medication, often dropping tablets unintentionally without noticing. Therefore, nurses addressing these patients' specific needs was crucial for supporting their well‐being and effectively managing the challenges associated with their health conditions (Uhrenfeldt and Høybye 2014).

## 6 Discussion

To our knowledge, this is the first systematic review of medication management across the perioperative pathway involving patients with cancer. The findings of eight studies were synthesised in this systematic review, focusing on the factors associated with management of regular medications. Researchers employed various methodologies, including chart reviews, observations and interviews in the studies. The analysis revealed significant associations between management of patients' regular medications and postoperative complications, such as prolonged hospital stays, readmissions, mortality, morbidity and postdischarge institutionalisation. In addition, it was shown that improving patient and health care professional collaboration, along with pharmacist‐led interventions, enhanced the quality of medication management across the perioperative pathway.

Understanding the association between regular medication use and postoperative outcomes is essential for doctors, nurses and pharmacists who collaborate in managing patients' medications before and after surgery. For instance, polypharmacy, defined by the concurrent use of multiple medications, shows varied but significant links to adverse postoperative outcomes, especially in older patients (Chen et al. 2021; Choi et al. 2018; Jeong et al. 2018). Hence, utilising polypharmacy as a predictor of postoperative complications to proactively prevent harm to patients would be advantageous. However, conflicting results regarding the influence of polypharmacy on postoperative complications were reported in the studies. The comparison is complicated by the heterogeneity in outcome measures, as researchers examined various outcomes, including postdischarge institutionalisation, mortality, unplanned readmission and postoperative delirium. Moreover, the retrospective cohort designs employed in these studies introduced variability in data collection and the handling of confounding variables. However, the study by Chen et al. (2021), with a large cohort of homogenous participants with the same cancer, and a more precise definition of polypharmacy, lends greater credibility to the association of excessive polypharmacy with poorer cancer survival. These associations emphasise the need for careful review of regular medications and optimisation before surgery. The conflicting results regarding postoperative complications suggest that the impact of polypharmacy may be influenced by other factors, such as the types of medications used and the underlying health status of the patients, like their cancer type (Jeon et al. 2020; Jeong et al. 2018). The potential influence of cancer type on postoperative complications was examined, showing significant association of gastrointestinal cancers with unplanned readmissions and prolonged hospital stay (Jeon et al. 2020; Jeong et al. 2018). These findings may be linked to the higher risk of complications associated with intra‐abdominal surgeries commonly performed for gastrointestinal cancers (Aust et al. 2005). In contrast, other studies that included patients from various cancer types did not find similar associations (Choi et al. 2018; Jeong et al. 2016), suggesting the need for further studies utilising controlled trial designs to establish this association or causality robustly in the perioperative environment.

Potentially inappropriate medication use seems to have significant impact on prolonged hospital stay among cancer patients undergoing surgery (Jeong et al. 2018; Samuelsson et al. 2016). Simultaneously, the conflicting findings regarding the association between potentially inappropriate medication use and mortality in the studies by Choi et al. (2018) and Samuelsson et al. (2016) can be explained by differences in study design and population. Samuelsson et al.'s study, which included a larger cohort, older participants (aged 75 years and over), elective and emergency surgeries and a higher incidence of mortality (368 deaths), provides more robust and credible results. These findings support the use of tools like the Beers Criteria (‘American Geriatrics Society 2019 Updated AGS Beers Criteria for Potentially Inappropriate Medication Use in Older Adults’, 2019) to identify and manage potentially inappropriate medications effectively.

There is limited evidence supporting an association between preoperative discontinuation‐requiring medications and postoperative complications. However, herbal extracts, which were considered as regular medications in Jeon et al.'s (2020) study, have been identified as a significant risk factor for readmission within 30 days postoperatively. This risk may be due to insufficient information about their effect on the body, as well as the common belief among patients that herbal extracts are safe. Consequently, many patients do not inform their doctors about using these medications, leading to inadequate management in the perioperative environment. In contrast, antithrombotic agents did not demonstrate a significant association with readmission, likely attributable to the meticulous management protocols employed for these medications in the perioperative period.

The evidence showed that collaborative practices between patients and health care professionals, as well as among health care professionals, significantly enhanced medication management throughout the perioperative pathway (Triscari, Teoh, and Femia 2022; Uhrenfeldt and Høybye 2014). The integration of pharmacists into the perioperative team, as demonstrated by Triscari, Teoh, and Femia (2022), exemplifies this collaborative practice among health care professionals. Pharmacists provide specialised knowledge that enhances the expertise of other health care professionals, particularly nurses, creating a multidisciplinary team capable of addressing the complex challenges of medication management in patients. Simultaneously, nurses' collaboration with patients in managing their medication consumption underscores collaborative practice between health care professionals and patients (Triscari, Teoh, and Femia 2022). By working together, health care professionals can ensure that medication plans are safe, effective and tailored to the individual needs of each patient.

### 6.1 Limitations of the Systematic Review

Despite using a comprehensive search strategy, only studies published in English were included in this systematic review, which may introduce publication bias. Studies that included both cancer and noncancer patients were excluded if the results were not reported specifically for cancer patients. Similarly, studies conducted in both surgical and medical settings were excluded if the results were not reported specifically for surgical settings. A limitation of this review is that four of the included studies (Choi et al. 2018; Jeon et al. 2020; Jeong et al. 2016, 2018) utilised the same database as their baseline information to conduct separate analyses with distinct outcome measures. While their inclusion allowed for a diverse exploration of different aspects of medication use and postoperative complications, the reliance on a shared cohort may introduce potential overlap in findings and limit the generalisability of the results. Furthermore, the majority of the included studies focused on participants aged 65 years and older, limiting the generalisability of the outcomes to younger populations. Only one study included a broader age range, highlighting the need for further research to understand the impact of regular medication management on younger patients.

### 6.2 Implications for Practice

Expanding the role of pharmacists in the preadmission process, as well as collaborative efforts between nurses and patients can lead to more comprehensive reviews of regular medications and targeted interventions, reducing the risk of medication‐related complications postoperatively. Enhancing nurses' medication knowledge through collaboration with pharmacists represents a valuable approach to improving the quality of medication management processes, given nurses' crucial role in medication administration. Health care systems should prioritise deeper integration of pharmacists and nurses' roles in regular medication management within perioperative care teams.

## 7 Conclusion

The current body of research highlights significant gaps in our understanding of the impact of insufficient regular medication management on postoperative outcomes. There is a need for more studies with controlled trial designs to provide robust data on the causality of this relationship. Furthermore, it is important to study the impact of health care professionals' collaboration among themselves and with patients on the regular medication management of patients with cancer. The existing evidence is particularly sparse regarding adult patients with cancer undergoing surgery, especially those under the age of 65. Addressing these gaps through well‐designed research will be essential for optimising medication use and improving outcomes for this specific patient population.

## Author Contributions

A.M., P.N. and E.M. formulated the research question and developed the review protocol. A.M. searched the literature. Following, A.M., P.N. and E.M. screened the studies. A.M. and P.N. conducted data extraction and EM validated the data extracted. A.M. and P.N. assessed the quality of the studies. A.M. conducted data synthesis and drafted the manuscript, which was reviewed by P.N. and E.M. All authors reviewed and approved the final version.

## Conflicts of Interest

The authors declare no conflicts of interest.

## Peer Review

The peer review history for this article is available at https://www.webofscience.com/api/gateway/wos/peer‐review/10.1111/jan.16759.

## Protocol Registration

PROSPERO 2022 CRD42022330160 https://www.crd.york.ac.uk/prospero/display_record.php?ID=CRD42022330160.

## Supporting information


Appendix S1.

Appendix S2.

Appendix S3.

Appendix S4.

Appendix S5.

Appendix S6.

## Data Availability

Data sharing is not applicable to this article as no new data were created or analysed in this study.
