# Supplementary material for: Medication Management of Patients With Cancer Undergoing Surgery From Preadmission to Discharge: A Mixed‐Methods Systematic Review
Source: J Adv Nurs. 2025 Jan 21;81(10):6155–68. doi: 10.1111/jan.16759 (PMC12460946; doi:10.1111/jan.16759)
Supplement: Supplementary file 3 — Appendix S3. [file JAN-81-6155-s003.docx]

Top of Form

**Medication management in patients with cancer undergoing surgery from preadmission to discharge**

**Search History**

| **Embase Second edition 16.02.2023** | Friday, June 10, 2022 3:36:02 AM |
| --- | --- |

| **#** | **Searches** | **Results** |
| --- | --- | --- |
| #100 | #99 AND [embase]/lim NOT ([embase]/lim AND [medline]/lim) | **188** |
| #99 | #31 AND #61 AND #88 AND #97 AND [humans]/lim AND [english]/lim | 307 |
| #98 | #31 AND #61 AND #88 AND #97 | 323 |
| #97 | #89 OR #90 OR #91 OR #92 OR #93 OR #94 OR #95 OR #96 [Cancer patients] | 5,716,275 |
| #96 | 'malignant neoplasm'/exp | 4,390,364 |
| #95 | 'neoplasm'/de | 584,649 |
| #94 | 'surgical oncology'/exp | 2,240 |
| #93 | Oncolog*:ab,ti | 352,063 |
| #92 | Tumor*:ab,ti | 2,324,297 |
| #91 | Malignan*:ab,ti | 963,171 |
| #90 | Neoplasm:ab,ti | 95,193 |
| #89 | ‘Cancer patient*’:ab,ti | 375,153 |
| #88 | #62 OR #63 OR #64 OR #65 OR #66 OR #67 OR #68 OR #69 OR #70 OR #71 OR #72 OR #73 OR #74 OR #75 OR #76 OR #77 OR #78 OR #79 OR #80 OR #81 OR #82 OR #83 OR #84 OR #85 OR #86 OR #87 [Preadmission to discharge] | 1,661,350 |
| #87 | 'clinical handover'/de | 2,200 |
| #86 | 'transitional care'/de | 5,019 |
| #85 | 'hospitalization'/de | 501,920 |
| #84 | 'hospital readmission'/de | 92,406 |
| #83 | 'discharge'/exp | 206 |
| #82 | 'admission'/exp | 300 |
| #81 | 'patient care'/de | 347,226 |
| #80 | 'transition of care'/exp | 21 |
| #79 | ‘Hand off*’:ab,ti | 1,033 |
| #78 | Handoff*:ab,ti | 3,092 |
| #77 | ‘Hand over*’:ab,ti | 1,774 |
| #76 | Handover*:ab,ti | 3,880 |
| #75 | Hospitali?ation:ab,ti | 304,351 |
| #74 | Hospitali?ed:ab,ti | 229,469 |
| #73 | ‘Care continuum’:ab,ti | 2,932 |
| #72 | ‘Continuum of care’:ab,ti | 4,383 |
| #71 | ‘Coordination of care’:ab,ti | 3,136 |
| #70 | ‘Care continuity’:ab,ti | 870 |
| #69 | ‘Care transition*’:ab,ti | 3,473 |
| #68 | ‘Continuity of care’:ab,ti | 11,498 |
| #67 | ‘Interface* of care’:ab,ti | 49 |
| #66 | ‘Transitional care’:ab,ti | 2,860 |
| #65 | discharge:ab,ti | 360,977 |
| #64 | admission:ab,ti | 410,024 |
| #63 | Preadmission:ab,ti | 4,825 |
| #62 | ‘Transition* of care’:ab,ti | 3,949 |
| #61 | #32 OR #33 OR #34 OR #35 OR #36 OR #37 OR #38 OR #39 OR #40 OR #41 OR #42 OR #43 OR #44 OR #45 OR #46 OR #47 OR #48 OR #49 OR #50 OR #51 OR #52 OR #53 OR #54 OR #55 OR #56 OR #57 OR #58 OR #59 OR #60 [Surgical patient] | 2,823,896 |
| #60 | 'perioperative period'/de | 62,052 |
| #59 | 'perioperative nursing'/de | 6,897 |
| #58 | 'perioperative medicine'/de | 586 |
| #57 | 'perioperative care'/de | 785 |
| #56 | 'preoperative period'/de | 69,045 |
| #55 | 'preoperative evaluation'/de | 163,404 |
| #54 | 'preoperative education'/de | 757 |
| #53 | 'preoperative care'/de | 50,419 |
| #52 | 'postoperative period'/de | 256,773 |
| #51 | 'postoperative care'/de | 112,168 |
| #50 | 'postanesthesia care'/de | 1,266 |
| #49 | 'surgical patient'/de | 56,070 |
| #48 | 'surgery'/de | 744,735 |
| #47 | ‘Surgical patient*’:ab,ti | 37,787 |
| #46 | ‘Post-operat*’:ab,ti | 176,359 |
| #45 | ‘Pre-operat*’:ab,ti | 77,614 |
| #44 | ‘Post-surgery’:ab,ti | 25,022 |
| #43 | ‘Pre-surgery’:ab,ti | 4,155 |
| #42 | ‘Recovery room’:ab,ti | 5,054 |
| #41 | ‘Post-operative unit’:ab,ti | 22 |
| #40 | ‘Post-anaesthetic care unit’:ab,ti | 190 |
| #39 | ‘Post-anesthetic care unit’:ab,ti | 197 |
| #38 | PACU:ab,ti | 5,176 |
| #37 | Perioperat*:ab,ti | 189,056 |
| #36 | ‘Operating room’:ab,ti | 44,622 |
| #35 | ‘Operating suite’:ab,ti | 583 |
| #34 | ‘Operating theat*’:ab,ti | 8,675 |
| #33 | ‘Surgical procedure*’:ab,ti | 150,316 |
| #32 | Surger*:ab,ti | 1,957,505 |
| #31 | #1 OR #2 OR #3 OR #4 OR #5 OR #6 OR #7 OR #8 OR #9 OR #10 OR #11 OR #12 OR #13 OR #14 OR #15 OR #16 OR #17 OR #18 OR #19 OR #20 OR #21 OR #22 OR #23 OR #24 OR #25 OR #26 OR #27 OR #28 OR #29 OR #30 [Medication management] | 206,629 |
| #30 | 'drug monitoring'/de | 58,499 |
| #29 | 'pharmaceutical care'/exp | 22,692 |
| #28 | 'medication adherence assessment'/de | 5 |
| #27 | 'medication compliance'/exp | 43,817 |
| #26 | 'medication therapy management'/de | 14,321 |
| #25 | "Medication discontinu*":ab,ti | 1,083 |
| #24 | ‘Medication withhold*’:ab,ti | 11 |
| #23 | ‘Medication compliance’:ab,ti | 3,245 |
| #22 | ‘Medication adherence’:ab,ti | 21,760 |
| #21 | ‘Medication governance’:ab,ti | 3 |
| #20 | ‘Medication safety’:ab,ti | 4,078 |
| #19 | ‘Medication support’:ab,ti | 133 |
| #18 | ‘Medication discrepanc*’:ab,ti | 917 |
| #17 | ‘Medication reconciliation’:ab,ti | 3,946 |
| #16 | ‘use of medication*’:ab,ti | 9,179 |
| #15 | ‘drug usage’:ab,ti | 2,978 |
| #14 | ‘Medication history’:ab,ti | 3,777 |
| #13 | ‘Medication review*’:ab,ti | 4,575 |
| #12 | ‘Medication care’:ab,ti | 107 |
| #11 | ‘Medication change*’:ab,ti | 3,190 |
| #10 | ‘Drug therapy management’:ab,ti | 321 |
| #9 | ‘Medication therapy management’:ab,ti | 1,775 |
| #8 | ‘Pharmacological care’:ab,ti | 169 |
| #7 | ‘Managing medication*’:ab,ti | 575 |
| #6 | ‘management of medicine*’:ab,ti | 148 |
| #5 | ‘Pharmaceutical care’:ab,ti | 6,368 |
| #4 | ‘medicine* management’:ab,ti | 1,357 |
| #3 | ‘medication use’:ab,ti | 35,048 |
| #2 | ‘drug management’:ab,ti | 1,547 |
| #1 | ‘Medication management’:ab,ti | 6,965 |

Bottom of Form
