# Supplementary material for: Medication Management of Patients With Cancer Undergoing Surgery From Preadmission to Discharge: A Mixed‐Methods Systematic Review
Source: J Adv Nurs. 2025 Jan 21;81(10):6155–68. doi: 10.1111/jan.16759 (PMC12460946; doi:10.1111/jan.16759)
Supplement: Supplementary file 4 — Appendix S4. [file JAN-81-6155-s006.docx]

**Medication management in patients with cancer undergoing surgery from preadmission to discharge**

| **APA PsycINFO Second edition 16.02.2023** | Friday, June 10, 2022 3:36:02 AM |
| --- | --- |

| **#** | **Searches** | **Results** |
| --- | --- | --- |
| S84 | S28 AND S47 AND S73 AND S83 | 18 |
| S83 | S74 OR S75 OR S76 OR S77 OR S78 OR S79 OR S80 OR S81 OR S82 [Cancer patients] | 70,800 |
| S82 | DE "Oncology" | 5,557 |
| S81 | DE "Terminal Cancer" | 1,598 |
| S80 | DE "Metastasis" | 529 |
| S79 | DE "Neoplasms" | 45,433 |
| S78 | TI Tumor* OR AB Tumor* | 16,542 |
| S77 | TI Oncolog* OR AB Oncolog* | 10,416 |
| S76 | TI Malignan* OR AB Malignan* | 7,426 |
| S75 | TI Neoplasm OR AB Neoplasm | 19,150 |
| S74 | TI “Cancer patient*” OR AB “Cancer patient*” | 15,580 |
| S73 | S48 OR S49 OR S50 OR S51 OR S52 OR S53 OR S54 OR S55 OR S56 OR S57 OR S58 OR S59 OR S60 OR S61 OR S62 OR S63 OR S64 OR S65 OR S66 OR S67 OR S68 OR S69 OR S70 OR S71 OR S72 [Preadmission to discharge] | 186,460 |
| S72 | DE "Hospitalized Patients" | 14,322 |
| S71 | DE "Hospitalization" | 23,239 |
| S70 | DE "Health Care Delivery" | 22,295 |
| S69 | DE "Health Care Services" | 48,519 |
| S68 | DE "Continuum of Care" | 2,264 |
| S67 | DE "Hospital Discharge" | 2,945 |
| S66 | DE "Hospital Admission" | 3,302 |
| S65 | TI “Hand off*” OR AB “Hand off*” | 144 |
| S64 | TI Handoff* OR AB Handoff* | 287 |
| S63 | TI “Hand over*” OR AB “Hand over*” | 271 |
| S62 | TI Handover* OR AB Handover* | 407 |
| S61 | TI Hospitali?ation OR AB Hospitali?ation | 25,537 |
| S60 | TI Hospitali?ed OR AB Hospitali?ed | 22,928 |
| S59 | TI “Care continuum” OR AB “Care continuum” | 561 |
| S58 | TI “Continuum of care” OR AB “Continuum of care” | 1,114 |
| S57 | TI “Coordination of care” OR AB “Coordination of care” | 579 |
| S56 | TI “Care continuity” OR AB “Care continuity” | 197 |
| S55 | TI “Care transition*” OR AB “Care transition*” | 609 |
| S54 | TI “Continuity of care” OR AB “Continuity of care” | 2,495 |
| S53 | TI “Transitional care” OR AB “Transitional care” | 386 |
| S52 | TI “Interface* of care” OR AB “Interface* of care” | 7 |
| S51 | TI “Transition* of care” OR AB “Transition* of care” | 632 |
| S50 | TI Discharge OR AB Discharge | 34,968 |
| S49 | TI Admission OR AB Admission | 41,404 |
| S48 | TI Preadmission OR AB Preadmission | 403 |
| S47 | S29 OR S30 OR S31 OR S32 OR S33 OR S34 OR S35 OR S36 OR S37 OR S38 OR S39 OR S40 OR S41 OR S42 OR S43 OR S44 OR S45 OR S46 [Surgical patient] | 41,120 |
| S46 | DE "Surgical Patients" | 3,344 |
| S45 | DE "Surgery" | 14,254 |
| S44 | TI “Surgical patient*” OR AB “Surgical patient*” | 1,324 |
| S43 | TI “Post-operat*” OR AB “Post-operat*” | 2,233 |
| S42 | TI “Pre-operat*” OR AB “Pre-operat*” | 985 |
| S41 | TI “Post-surgery” OR AB “Post-surgery” | 970 |
| S40 | TI “Pre-surgery” OR AB “Pre-surgery” | 200 |
| S39 | TI “Recovery room” OR AB “Recovery room” | 80 |
| S38 | TI “Post-operative unit” OR AB “Post-operative unit” | 1 |
| S37 | TI “Post-anaesthetic care unit” OR AB “Post-anaesthetic care unit” | 3 |
| S36 | TI “Post-anesthetic care unit” OR AB “Post-anesthetic care unit” | 3 |
| S35 | TI PACU OR AB PACU | 54 |
| S34 | TI Perioperat* OR AB Perioperat* | 1,340 |
| S33 | TI “Operating room” OR AB “Operating room” | 558 |
| S32 | TI “Operating suite” OR AB “Operating suite” | 4 |
| S31 | TI “Operating theat*” OR AB “Operating theat*” | 206 |
| S30 | TI “Surgical procedure*” OR AB “Surgical procedure*” | 2,129 |
| S29 | TI Surger* OR AB Surger* | 31,829 |
| S28 | S1 OR S2 OR S3 OR S4 OR S5 OR S6 OR S7 OR S8 OR S9 OR S10 OR S11 OR S12 OR S13 OR S14 OR S15 OR S16 OR S17 OR S18 OR S19 OR S20 OR S21 OR S22 OR S23 OR S24 OR S25 OR S26 OR S27 [Medication management] | 162,162 |
| S27 | DE "Prescription Drugs" | 6,701 |
| S26 | DE "Prescribing (Drugs)" | 4,166 |
| S25 | DE "Polypharmacy" | 1,860 |
| S24 | DE “Drug Therapy” | 147,437 |
| S23 | TI “Medication discontinu*” OR AB “Medication discontinu*” | 219 |
| S22 | TI “Medication compliance” OR AB “Medication compliance” | 833 |
| S21 | TI “Medication adherence” OR AB “Medication adherence” | 4,702 |
| S20 | TI “Medication safety” OR AB “Medication safety” | 259 |
| S19 | TI “Medication support” OR AB “Medication support” | 33 |
| S18 | TI “Medication discrepanc*” OR AB “Medication discrepanc*” | 34 |
| S17 | TI “Medication reconciliation” OR AB “Medication reconciliation” | 140 |
| S16 | TI “use of medication*” OR AB “use of medication*” | 2,484 |
| S15 | TI “drug usage” OR AB “drug usage” | 621 |
| S14 | TI “Medication history” OR AB “Medication history” | 262 |
| S13 | TI “Medication review*” OR AB “Medication review*” | 315 |
| S12 | TI “Medication care” OR AB “Medication care” | 13 |
| S11 | TI “Medication change*” OR AB “Medication change*” | 347 |
| S10 | TI “Drug therapy management” OR AB “Drug therapy management” | 22 |
| S9 | TI “Medication therapy management” OR AB “Medication therapy management” | 69 |
| S8 | TI “Pharmacological care” OR AB “Pharmacological care” | 20 |
| S7 | TI “Managing medication*” OR AB “Managing medication*” | 137 |
| S6 | TI “management of medicine*” OR AB “management of medicine*” | 44 |
| S5 | TI “Pharmaceutical care” OR AB “Pharmaceutical care” | 166 |
| S4 | TI “medicine* management” OR AB “medicine* management” | 111 |
| S3 | TI “medication use” OR AB “medication use” | 5,017 |
| S2 | TI “drug management” OR AB “drug management” | 121 |
| S1 | TI “Medication management” OR AB “Medication management” | 1,664 |
