# Supplementary material for: Medication Management of Patients With Cancer Undergoing Surgery From Preadmission to Discharge: A Mixed‐Methods Systematic Review
Source: J Adv Nurs. 2025 Jan 21;81(10):6155–68. doi: 10.1111/jan.16759 (PMC12460946; doi:10.1111/jan.16759)
Supplement: Supplementary file 6 — Appendix S6. [file JAN-81-6155-s005.pdf]

Systematic review

Please select one of the options below to edit your record. Either option will create a new version of the record - the existing version will remain unchanged.

A list of fields that can be edited in an update can be found here

1. * Review title.

Give the title of the review in English

Medication management in patients with cancer undergoing surgery from preadmission to discharge

2. Original language title.

For reviews in languages other than English, give the title in the original language. This will be displayed with the English language title.

3. * Anticipated or actual start date.

Give the date the systematic review started or is expected to start.

30/11/2022

4. * Anticipated completion date. [1 change]

Give the date by which the review is expected to be completed.

31/03/2025

5. * Stage of review at time of this submission. [2 changes]

This field uses answers to initial screening questions. It cannot be edited until after registration.

Tick the boxes to show which review tasks have been started and which have been completed.

Update this field each time any amendments are made to a published record.

The review has not yet started: No

Review stage	Started	Completed
Preliminary searches	No	Yes
Piloting of the study selection process	Yes	Yes
Formal screening of search results against eligibility criteria	Yes	Yes
Data extraction	Yes	Yes
Risk of bias (quality) assessment	Yes	Yes

Review stage	Started	Completed
Data analysis	Yes	Yes

Provide any other relevant information about the stage of the review here.

6. * Named contact.

The named contact is the guarantor for the accuracy of the information in the register record. This may be any member of the review team.

Atefeh Mehrabifar

Email salutation (e.g. "Dr Smith" or "Joanne") for correspondence:
Atefeh

7. * Named contact email.

Give the electronic email address of the named contact.

s218600423@deakin.edu.au

8. Named contact address

PLEASE NOTE this information will be published in the PROSPERO record so please do not enter private information, i.e. personal home address

Give the full institutional/organisational postal address for the named contact.

School of Nursing and Midwifery, Faculty of Health, Burwood Campus, Deakin University

9. Named contact phone number.

Give the telephone number for the named contact, including international dialling code.

+61 3 92468836 ext:68836

10. * Organisational affiliation of the review.

Full title of the organisational affiliations for this review and website address if available. This field may be completed as 'None' if the review is not affiliated to any organisation.

School of Nursing and Midwifery, Faculty of Health, Deakin University

Organisation web address:
https://www.deakin.edu.au

11. * Review team members and their organisational affiliations.

Give the personal details and the organisational affiliations of each member of the review team. Affiliation refers to groups or organisations to which review team members belong.
NOTE: email and country now MUST be entered for each person, unless you are amending a published record.
PLEASE USE AN INSTITUTIONAL EMAIL ADDRESS IF POSSIBLE.

Mrs Atefeh Mehrabifar. School of Nursing and Midwifery, Faculty of Health, Deakin University

Assistant/Associate Professor Patricia Nicholson. School of Nursing and Midwifery, Faculty of Health, Deakin University

Professor Elizabeth Manias. School of Nursing and Midwifery, Faculty of Medicine, Nursing and Health Sciences, Monash University

12. * Funding sources/sponsors.

Details of the individuals, organizations, groups, companies or other legal entities who have funded or sponsored the review.

Deakin University Postgraduate Research Scholarship (DUPRS)

Grant number(s)

State the funder, grant or award number and the date of award

Deakin University Postgraduate Research Scholarship (DUPRS) includes the cost of tuition fees and provides a living allowance (March 2022 for 3 years). (00113B)

13. * Conflicts of interest.

List actual or perceived conflicts of interest (financial or academic).

None

14. Collaborators.

Give the name and affiliation of any individuals or organisations who are working on the review but who are not listed as review team members. **NOTE: email and country must be completed for each person, unless you are amending a published record.**

15. * Review question.

State the review question(s) clearly and precisely. It may be appropriate to break very broad questions down into a series of related more specific questions. Questions may be framed or refined using PI(E)COS or similar where relevant.

How are medications managed among patients with cancer undergoing surgery from preadmission to discharge?

16. * Searches. [1 change]

State the sources that will be searched (e.g. Medline). Give the search dates, and any restrictions (e.g. language or publication date). Do NOT enter the full search strategy (it may be provided as a link or attachment below.)

Databases including PubMed, MEDLINE, CINAHL, Embase, Cochrane Library, Informit Health Collection, and PsycINFO will be searched for relevant studies. Cross-checking of citations, and references will be conducted in Scopus and Web of Science databases to find further relevant studies. Regarding the time limit, literature published from inception until November of 2022 will be included in the search. All available English language materials will be examined (Search updated in February 2023)

17. URL to search strategy.

Upload a file with your search strategy, or an example of a search strategy for a specific database, (including the keywords) in pdf or word format. In doing so you are consenting to the file being made publicly accessible.

Or provide a URL or link to the strategy. Do NOT provide links to your search **results**.

Do not make this file publicly available until the review is complete

18. * Condition or domain being studied.

Give a short description of the disease, condition or healthcare domain being studied in your systematic review.

The medication management of patients with cancer undergoing surgery from preadmission to discharge

19. * Participants/population.

Specify the participants or populations being studied in the review. The preferred format includes details of both inclusion and exclusion criteria.

Inclusion: Adult patients aged 18 years and over, with cancer undergoing surgery
Exclusion: Day surgery

20. * Intervention(s), exposure(s).

Give full and clear descriptions or definitions of the interventions or the exposures to be reviewed. The preferred format includes details of both inclusion and exclusion criteria.

Medication management is defined as optimum medication use in patients while minimising the risk of medication-related problems and costs imposed on patients and health care system. This process includes medication history taking, medication reconciliation, medication review, and all pharmaceutical services when patients are hospitalised. In this review, all medication management processes will be reviewed in different stages, from prescribing, preparing, administering to monitoring.

21. * Comparator(s)/control.

Where relevant, give details of the alternatives against which the intervention/exposure will be compared (e.g. another intervention or a non-exposed control group). The preferred format includes details of both inclusion and exclusion criteria.

Not applicable

22. * Types of study to be included.

Give details of the study designs (e.g. RCT) that are eligible for inclusion in the review. The preferred format includes both inclusion and exclusion criteria. If there are no restrictions on the types of study, this should be stated.

Any study designs will be included; qualitative, quantitative (retrospective, prospective, cross-sectional, longitudinal, case-control and cohort studies, and randomised controlled trial), and mixed methods will be eligible for inclusion.

23. Context.

Give summary details of the setting or other relevant characteristics, which help define the inclusion or exclusion criteria.

Studies in the perioperative environments and surgical wards in metropolitan sites, or in regional, rural or remote areas.

24. * Main outcome(s).

Give the pre-specified main (most important) outcomes of the review, including details of how the outcome is defined and measured and when these measurement are made, if these are part of the review inclusion criteria.

To understand how patients' transition from preadmission to discharge influence medication management.

Measures of effect
Regarding interventional, prospective, and retrospective cohort studies, we will examine them for any commonalities of outcomes, for example, reduction in medication errors across clinical settings. This information will be analysed using odds ratios and mean differences.

25. * Additional outcome(s).

List the pre-specified additional outcomes of the review, with a similar level of detail to that required for main outcomes. Where there are no additional outcomes please state 'None' or 'Not applicable' as appropriate to the review

None

Measures of effect
None

26. * Data extraction (selection and coding).

Describe how studies will be selected for inclusion. State what data will be extracted or obtained. State how this will be done and recorded.

Two reviewers will apply eligibility criteria and select studies for inclusion in the systematic review. This review will be done at the abstract and full-text levels and recorded using Covidence software. All records for inclusion will be screened independently and blinded to each other's' decisions. A third independent reviewer will check the reviewers' decisions and any conflicts they might have. The disagreements will be resolved through consensus.

The data to be extracted would include the study design, methodology, and participants. Two reviewers will extract the data; a third independent reviewer will check the reviewers' decisions and any conflicts. The disagreements will be resolved through consensus.

.

27. * Risk of bias (quality) assessment. [1 change]

State which characteristics of the studies will be assessed and/or any formal risk of bias/quality assessment tools that will be used.

For quantitative studies, methods of randomisation, data collection process and results will be appraised in quality assessment. As for the qualitative studies, the methodology and research findings will be evaluated. Two reviewers will evaluate the quality of the studies by using the MIXED METHODS APPRAISAL TOOL (MMAT), VERSION 2018, and a third reviewer will check the two researchers' decisions.

To assess agreement, the data will be reviewed in a meeting, and any disagreement will be resolved through consensus.

28. * Strategy for data synthesis.

Describe the methods you plan to use to synthesise data. This **must not be generic text** but should be **specific to your review** and describe how the proposed approach will be applied to your data.
If meta-analysis is planned, describe the models to be used, methods to explore statistical heterogeneity, and software package to be used.

The quantitative data will be synthesised based on the degree of consistency in findings and the measured variables relevant to the review question. If the data are sufficiently homogenous, a meta-analysis will be conducted. Otherwise, the data will be synthesised using narrative or descriptive synthesis. This process will be checked by another reviewer.

The qualitative studies will be synthesised through a meta-synthesis. In the first step, primary qualitative studies will be read line by line to identify and code the findings relevant to the review question. This process will be checked by another reviewer. Following, these data will be examined to develop descriptive themes based on their meanings. Next, they will be grouped based on their similarities and differences to generate a set of statements beyond the original studies' findings on the phenomena.

29. * Analysis of subgroups or subsets.

State any planned investigation of 'subgroups'. Be clear and specific about which type of study or participant will be included in each group or covariate investigated. State the planned analytic approach.

The studies will be categorised based on the study design such as qualitative studies, quantitative studies, and mixed methods studies. The subgroups will be formed based on the stage of the patient care from preadmission to discharge that the patient's medication management was investigated or based on the outcome measure in the studies.

30. * Type and method of review.

Select the type of review, review method and health area from the lists below.

Type of review

Cost effectiveness	No
Diagnostic	No
Epidemiologic	No
Individual patient data (IPD) meta-analysis	No
Intervention	No
Living systematic review	No
Meta-analysis	Yes
Methodology	No
Narrative synthesis	Yes
Network meta-analysis	No
Pre-clinical	No
Prevention	No
Prognostic	No
Prospective meta-analysis (PMA)	No
Review of reviews	No
Service delivery	No
Synthesis of qualitative studies	Yes
Systematic review	Yes
Other	No

Health area of the review

Alcohol/substance misuse/abuse	No
Blood and immune system	No
Cancer	Yes
Cardiovascular	No
Care of the elderly	No
Child health	No
Complementary therapies	No

COVID-19	No
Crime and justice	No
Dental	No
Digestive system	No
Ear, nose and throat	No
Education	No
Endocrine and metabolic disorders	No
Eye disorders	No
General interest	No
Genetics	No
Health inequalities/health equity	No
Infections and infestations	No
International development	No
Mental health and behavioural conditions	No
Musculoskeletal	No
Neurological	No
Nursing	No
Obstetrics and gynaecology	No
Oral health	No
Palliative care	No
Perioperative care	Yes
Physiotherapy	No
Pregnancy and childbirth	No
Public health (including social determinants of health)	No
Rehabilitation	No
Respiratory disorders	No
Service delivery	Yes

Skin disorders	No
Social care	No
Surgery	Yes
Tropical Medicine	No
Urological	No
Wounds, injuries and accidents	No
Violence and abuse	No

31. Language.

Select each language individually to add it to the list below, use the bin icon to remove any added in error.

English

There is an English language summary.

32. * Country.

Select the country in which the review is being carried out. For multi-national collaborations select all the countries involved.

Australia

33. Other registration details.

Name any other organisation where the systematic review title or protocol is registered (e.g. Campbell, or The Joanna Briggs Institute) together with any unique identification number assigned by them.
If extracted data will be stored and made available through a repository such as the Systematic Review Data Repository (SRDR), details and a link should be included here. If none, leave blank.

Deakin University's research repository

34. Reference and/or URL for published protocol.

If the protocol for this review is published provide details (authors, title and journal details, preferably in Vancouver format)

No I do not make this file publicly available until the review is complete

35. Dissemination plans.

Do you intend to publish the review on completion?

Yes

36. Keywords.

Give words or phrases that best describe the review. Separate keywords with a semicolon or new line. Keywords help PROSPERO users find your review (keywords do not appear in the public record but are included in searches). Be as specific and precise as possible. Avoid acronyms and abbreviations unless these are in wide use.

Systematic review, medication management, surgery, cancer patients.

37. Details of any existing review of the same topic by the same authors.

If you are registering an update of an existing review give details of the earlier versions and include a full bibliographic reference, if available.

NA

38. * Current review status. [1 change]

Update review status when the review is completed and when it is published.
New registrations must be ongoing so this field is not editable for initial submission.

Review_Completed_not_published

39. Any additional information.

Provide any other information relevant to the registration of this review.

This review will be undertaken from 30.11.2022 to 30.05.2024.
This review is being undertaken as part of a PhD at Deakin University

40. Details of final report/publication(s) or preprints if available.

Leave empty until publication details are available OR you have a link to a preprint (NOTE: this field is not editable for initial submission).
List authors, title and journal details preferably in Vancouver format.
